# Anthocyanin Structure and pH Dependent Extraction Characteristics from Blueberries (*Vaccinium corymbosum*) and Chokeberries (*Aronia melanocarpa*) in Subcritical Water State

**DOI:** 10.3390/foods10030527

**Published:** 2021-03-03

**Authors:** Hye-Ji Kang, Min-Jung Ko, Myong-Soo Chung

**Affiliations:** 1 Department of Food Science and Engineering, Ewha Womans University, Seoul 03760, Korea; kjskai2013@naver.com; 2 Department of Food Science and Biotechnology, Global K-Food Research Center, Hankyong National University, Anseong-si 17579, Korea; mjko@hknu.ac.kr

**Keywords:** blueberries, chokeberries, subcritical-water extraction (SWE), anthocyanin structure

## Abstract

This study determines the optimal extraction conditions for the subcritical-water extraction (SWE) of anthocyanin from blueberries and chokeberries and compares the performance using conventional extraction methods. SWE is carried out under different conditions of extraction temperature (110 °C, 130 °C, 150 °C, 170 °C, 190 °C, and 200 °C), extraction time (1, 3, 5, and 10 min), and solvent pH (water and 1% citric acid). The solubility and stability of anthocyanin from blueberries and chokeberries influences the optimal condition for SWE. The presence of more methoxy and hydroxyl functional groups in the basic skeleton of anthocyanin will result in a lower solubility at a high temperature. Water at a higher temperature exhibits a better dissociation reaction, and a solvent has a lower pH at a higher temperature. One percent citric acid is used to reduce the pH of the solvent, which increases the extraction efficiency of anthocyanin in a subcritical water state.

## 1. Introduction

Anthocyanidin is an aglycon form of anthocyanin, which is a colored phenolic compound that exists in nature. The color of anthocyanidin varies with the positions and types of the attached functional groups: as the number of attached functional groups such as hydroxyl and methoxy groups increases, the color of anthocyanidin changes from orange to purple. Anthocyanin pigments are known to exert various health effects, such as high antioxidant activity, reduction of low-density lipoprotein cholesterol, and lowering of arterial stiffness [1,2,3,4].

Anthocyanins in plants are mainly distributed in the vacuoles that are present inside cell walls, and are abundant in fruits, vegetables, and flowers [5]. Among fruits, blueberries (*Vaccinium corymbosum*) content is dominated by 41.4% malvidin glycoside, then 21.7% delphinidin glycoside, 19.23% petunidin glycoside, 12.4% peonidin glycoside, and 5.2% cyanidin glycoside. Chokeberries (*Aronia melanocarpa*) contain the highest amount of anthocyanins, overwhelmingly dominated by cyanidin glycoside (98.4%), then 0.08% petunidin glycoside, 0.3% malvidin glycoside, 0.3% pelargonidin glycoside, and 0.2% delphinidin glycoside [6]. Anthocyanin is sensitive to temperature, light, pH, oxygen, metal ions, and enzymes [7]. Occurring at high temperatures, the C-ring structure is destroyed after removing sugar from anthocyanin [8]. The structure of the anthocyanin also changes with the proton concentration, resulting in anthocyanin being reddish at pH 1, blue at pH 2–4, and colorless at pH 5 or higher [9].

The use of water as a solvent below 100 °C at atmospheric pressure makes it easy to extract polar compounds due to its high dielectric constant (ε) (ε < 80), but this is not effective for extracting nonpolar phenolic compounds or flavonoids. Subcritical-water extraction (SWE) can overcome these drawbacks, since the dielectric constant of water can be reduced (e.g., from ε = 53 at 110 °C to ε = 34.5 at 200 °C) below the critical point by applying appropriate pressure and temperature (100–374 °C). The use of converted subcritical water makes it easy to extract less polar compounds and is also environmentally friendly because no organic solvent is used. Previous studies have extracted components such as phenolic compounds and flavonoids using the SWE method [10,11]. Since SWE involves only using water and is cost-effective, rapid, and efficient, it has been applied to extract anthocyanins from plants and fruits, such as the anthocyanins from grape skins and from freeze-dried Sunbelt red grape pomace [12,13]. However, SWE has not previously been applied to characteristics of anthocyanin structures and is pH dependent during the subcritical state of blueberries, and chokeberries.

This study applies the SWE method to extract anthocyanin from blueberries and chokeberries, with the aim of determining the optimum condition such as the time, temperature, and pH of the solvent for maximizing its extraction efficiency, and their dependence on the chemical structure of anthocyanins. Furthermore, the anthocyanin concentration in the optimum condition is compared with that for conventional extraction methods. The flavonoid content and antioxidant activity of the extracts are evaluated. The pH differential method and high-performance liquid chromatography (HPLC) are performed to analyze the blueberry and chokeberry extracts, and the correlation between the findings of these two methods also is analyzed.

## 2. Materials and Methods

### 2.1. Materials

Frozen blueberries (14 mm, about 3 g), and chokeberries (14 mm, about 2.8 g) were obtained from Pyeongtaek and Gochang in Korea. These berries were harvested in 2019. Samples were dried for 24 h using a freeze dryer (FD8523, Ilshin, Gyeonggi-do, Korea). The obtained samples dried to a moisture content of <20% were passed through a 20-mesh sieve and stored in the dark at −4 °C prior to performing the extraction process.

Anthocyanins are commonly unstable to processing and storage. Previous studies regarding anthocyanin stability in frozen storage up to 4 months found it did not significantly affect the anthocyanins of berries [14] and grinding for extraction had positive effects on the anthocyanin yield due to the increase in surface area [15]. However, a high temperature for sample drying negatively influenced anthocyanin stability [14,16]. Therefore, samples of blueberries and chokeberries were dried using a freeze dryer in this study.

### 2.2. Chemicals and Reagents

Standard malvidin-3-galactoside chloride (molecular weight = 528.89, purity grade ≥ 95%), and cyanidin-3-galactoside chloride (molecular weight = 484.84, purity grade ≥ 95%) were purchased from Sigma-Aldrich (Yongin, Gyunggi-do, Korea) and Dongmyung Scientific (Seoul, Korea). CH_2_O_2_ (formic acid), C_2_H_4_O_2_ (glacial acetic acid), CH_3_CN (acetonitrile), and H_3_PO_4_ (phosphoric acid) of an analytical grade were purchased from Duksan (Ansan, Gyeonggi-do, Korea). Water and CH_3_OH (methanol; high-performance liquid chromatography (HPLC) grade) were obtained from J.T. Baker (Phillipsburg, NJ, USA). The buffer was made using HCl (hydrogen chloride), KCl (potassium chloride), and C_2_H_3_NaO_2_ (sodium acetate) obtained from Duksan.

### 2.3. Extraction Methods

The subcritical-water extraction (SWE) process was performed using an accelerated solvent extractor (ASE 350, Dionex, Sunnyvale, CA, USA) with Milli-Q water (MR-RO800, Mirae ST, Anyang, Korea). The dried sample (1 g) and diatomaceous earth (2 g, Dionex) were placed in a stainless-steel extraction cell (23 mm i.d. × 50 mm long, 22 mL; Dionex) containing a cellulose filter. Extraction was performed under various temperatures (110–200 °C) and times (1–10 min). Subcritical solvent extraction also was performed using 1% citric acid as the subcritical solvent under the SWE optimum conditions (130 °C for 3 min for blueberries, 190 °C for 1 min for chokeberries).

The accelerated solvent extractor was set up in the bypass heat-up mode, and a rinse process was carried out between successive extractions. The extraction process was as follows: The sample cell was loaded into the oven once it had reached the pre-set temperature. The pump was then operated to fill the cell with solvent and, once the cell contents had reached the set temperature, it was maintained for the set extraction time under constant pressure (about 100 bar). Once the extraction time had elapsed, the extract from the cell was transferred to the collection bottle along the line through the filter using nitrogen gas.

To confirm the anthocyanin extraction efficiency of the SWE method, conventional extraction methods were applied by pressing blueberry and chokeberry juice and applying a hot-water extraction method. The pressed-juice extract was prepared by thawing frozen blueberries and chokeberries at room temperature (25 °C), applying pressure, and then extracting the juice after screening. Hot-water extraction was performed by mixing 1 g of a powder sample and 22 mL of distilled water (DW) in a water bath maintained at 60 °C for 1 h. The extraction processes were performed in the dark, and all experiments were conducted in triplicate.

The extracted blueberry and chokeberry extracts were kept in a deep freezer at −80 °C for more than 12 h and then dried for 24 h using a freeze dryer. The dried extract was dissolved in 15 mL of methanol and vortexed, and then centrifugation was performed at 4000 rpm for 20 min to separate the solid and supernatant. The supernatant was passed through a 0.45-μm 13-mm-long polyvinylidene fluoride (PVDF) filter (Syringe Filter, Whatman, NJ, USA) before being analyzed.

### 2.4. Determination of Total Anthocyanin Contents

The anthocyanin content was determined using the pH differential method. The color of anthocyanin varies with the pH, being reddish at pH 1 and colorless at pH 4.5, so samples were diluted in buffers at pH 1 (prepared using HCl and sodium acetate) and pH 4.5 (prepared using HCl and KCl). A 0.1-mL aliquot of each sample was added to 1.4 mL of each buffer and vortexed. The absorbance was then measured at 510 and 700 nm with a spectrophotometer to predict the anthocyanin content using the following formula:

(1) $$\mathrm{Anthocyanin}\;(\mathrm{mg}/L)\;=\;\frac{A\;\times \;1000\;\times \;\mathrm{MW}\;\times \;\mathrm{DF}}{\varepsilon \;\times \;1}$$

where A = (A_510 nm_ − A_700 nm_ at pH 1) − (A_510 nm_ − A_700 nm_ at pH 4.5), MW = molecular weight of standard anthocyanin, DF = dilution factor, l = path length (in cm), ε = molar extinction coefficient (in L/mol/cm), and 1000 = factor for converting from grams to milligrams [17]. 

### 2.5. High-Performance Liquid Chromatography (HPLC) and Mass Spectrometry (MS)

The extracts were simply pretreated and quantitatively analyzed by high-performance liquid chromatography (HPLC) (1260 Infinity, Agilent Technologies, Waldbronn, Germany) with a Zorbax C18 column (4.6 mm × 100 mm, 5 μm pore size; Agilent Technologies, Waldbronn, Germany). The HPLC analysis method followed a modified version of the method reported by Kalt et al. [18] for analyzing blueberry extracts. The mobile phase used for analysis comprised 5% aqueous formic acid (solvent A) and methanol (solvent B). The percentage of solvent B was increased from 14% to 17% over 10 min, then to 23% over 25 min, then to 47% over 30 min, then to 14% over 2 min, and then to 14% over 3 min. The injection volume was 50 μL and the flow rate was 1 mL/min. Detection was performed at 515 nm using a Ultraviolet (UV) detector (Agilent Technologies, Waldbronn, Germany). The HPLC analysis method followed a modified version of the method reported by Chandra et al. [19] for analyzing chokeberry extracts. The mobile phase used for analysis comprised 0.5% aqueous phosphoric acid (solvent A) and water:acetonitrile:glacial acetic acid:phosphoric acid at 50:48.5:1.0:0.5 (*v*/*v*/*v*/*v*) (solvent B). The percentage of solvent B was increased from 0 to 20% over 26 min, then to 60% over 4 min, and then to 20% over 5 min. The injection volume was 10 μL and the flow rate was 0.8 mL/min. Detection was performed at 520 nm using a UV detector.

HPLC mass spectrometry (MS) was performed using a quadrupole time-of-flight mass spectrometer (6530 Accurate-Mass Q-TOF, Agilent Technologies, Waldbronn, Germany) equipped with an electrospray ionization source. The analysis parameters were set using positive-ion modes. The mobile phase used for the analysis of the chokeberry extracts comprised 0.1% formic acid in water (solvent A) and 0.1% formic acid in acetonitrile (solvent B). The injection volume was 5 μL and flow rate was 0.4 mL/min, with an initial solvent A:solvent B ratio of 90:10. The ratio of solvent B was changed as follows: to 12% over 8 min, to 15% over 10 min, to 15% over 15 min, to 55% over 18 min, to 90% over 20 min, and to 5% over 22 min.

The extracts were quantitatively identified by HPLC, and more accurately qualitatively analyzed by HPLC mass spectrometry.

### 2.6. Total Flavonoid Content

The AlCl_3_ (aluminum chloride) colorimetric method was used to determine the total flavonoid content. The sample (1 mL) was mixed with 0.3 mL of NaNO_2_ (sodium nitrite; 5%, *w*/*v*) and reacted for 5 min. Subsequently, 0.5 mL of AlCl_3_ (2%, *w*/*v*) was added and reacted for 6 min. Finally, 0.5 mL of 1 M NaOH (sodium hydroxide) was added and reacted for 10 min and the absorbance was then measured at 510 nm. Catechin was used to obtain a standard curve and the total flavonoid contents were expressed in milligrams of catechin equivalent (CE) per gram fresh weight (FW).

### 2.7. Antioxidant Activity

The ferric-reducing antioxidant power (FRAP) assay was used to measure the antioxidant activity of the extracts. The reagents were prepared for the FRAP assay as follows: To prepare the 300 mM acetate buffer, 3.1 g of C_2_H_3_NaO_2_·3H_2_O (sodium acetate trihydrate) was added to 16 mL of C_2_H_4_O_2_ (glacial acetic acid), with distilled water (DW) added to bring the total volume to 1 L. The 2,4,6-tri(2-pyridyl)-s-triazine (TPTZ) solution (10 mM) was prepared by adding 3.1233 g of TPTZ to 1 L of 40 mM HCl, and the FeCl_3_·6H_2_O solution was prepared by adding 1 L of DW to 5.406 g of FeCl_3_·6H_2_O. The working FRAP reagent was made by mixing the 300 mM acetate buffer, 10 mM TPTZ, and FeCl_3_·6H_2_O solution at a ratio of 10:1:1, and then preheated at 37 °C. The sample (10 μL) and 300 μL of working FRAP reagent were reacted on a 96-well plate and incubated for 5 min at 37 °C in the dark. The absorbance was measured at 593 nm. Trolox was used to obtain a standard curve, and the antioxidant activity was expressed in milligrams of trolox equivalent (TE) per gram fresh weight (FW).

### 2.8. Scanning Electron Microscopy

Images of the chokeberry powder were obtained using an environmental scanning electron microscopy (SEM; FEI XL-30FEG, FEI, Burlington, VT, USA) at the Korea Institute of Science and Technology.

### 2.9. Data Analysis

Pearson’s correlation coefficients were calculated as were Tukey’s test, and a one-way analysis of variance with Duncan’s test applied using SPSS Statistics software (version 22, IBM SPSS, IL, USA) with a probability cutoff for statistical significance of *p* < 0.05.

## 3. Results and Discussion

### 3.1. Results from pH Differential Method and High-Performance Liquid Chromatography (HPLC) Analysis

The malvidin-3-galactoside and cyanidin-3-galactoside standards for high-performance liquid chromatography (HPLC) analysis were diluted in methanol at concentrations of 0.03125, 0.0625, 0.125, 0.25, 0.5, and 1 mg/mL. The standard curves y = 35650x − 270.83 (R^2^ = 0.99) for malvidin-3-galactoside, and y = 28550x − 774.86 (R^2^ = 0.99) for cyanidin-3-galactoside were obtained. All the extracts obtained from blueberries and chokeberries were analyzed by HPLC; their HPLC chromatograms are shown in Figure S1.

A preliminary experiment was conducted to set the measurement wavelength to reduce the measurement error of the pH differential method. Extraction samples were loaded into the pH 1 and pH 4.5 buffers and the absorbance was measured across all wavelengths. This revealed that the absorbance difference between the two buffers was largest at 510 nm, with no difference observed at 700 nm (Figure S2). Therefore, the measured wavelengths were set as 510 and 700 nm.

The pH differential method is an analytical method based on the color of anthocyanin varying with pH, whereas the HPLC method predicts the content in a sample using a standard [17]. This study analyzed the blueberry and chokeberry extracts using both the pH differential method and the HPLC method, and the correlation between the pH differential method and the HPLC method also was analyzed (Figure S3). The correlation coefficient for the blueberry and chokeberry samples were 0.967, and 0.986, respectively. Although the strong correlation indicated that the two analytical methods showed similar tendencies, the content was lower when using the pH differential method than when using the HPLC method. The pH differential method is known to be less accurate than the HPLC method, but the cost of the anthocyanin standard is very high due to the difficulty in purifying it. This situation prompted the development of the pH differential method, which can be used to easily estimate the anthocyanin content using a spectrophotometer in a laboratory without requiring an anthocyanin standard.

The following factors contributed to the different results obtained when using the two methods: the moisture content, the hygroscopicity of the anthocyanins, the presence of impurities (polyphenolics or polymeric anthocyanins), the molar extinction coefficient, and the measured absorbance values. These factors resulted in the content measured using the pH differential method generally being lower than that for HPLC. A previous study by Lee et al. [17] also found a difference in the anthocyanin content between the pH differential method (3.56 mg/100 mL) and HPLC analysis (14.09 mg/100 mL).

### 3.2. Optimal Conditions of Subcritical-Water Extraction (SWE) from Blueberry and Chokeberry

**Figure 1:**
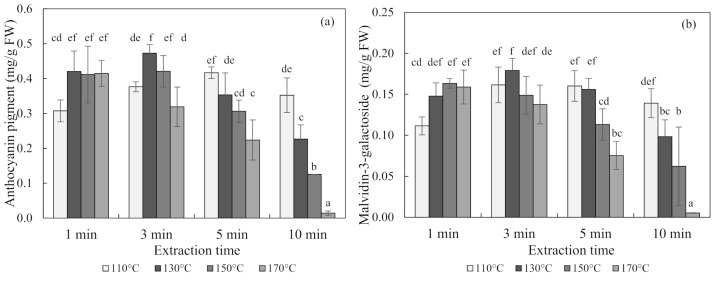
Anthocyanin pigment (**a**) and malvidin-3-galactoside (**b**) concentrations of blueberry extracts obtained using subcritical-water extraction (SWE) as analyzed by the pH differential method and high-performance liquid chromatography (HPLC) analysis. Means in bars with same letters are not significantly different according to Duncan’s test at *p* < 0.05. Data are mean and SD values (*n* = 3). FW (fresh weight)

The contents of anthocyanin in blueberries obtained using subcritical-water extraction (SWE) at 110 °C, 130 °C, 150 °C, and 170 °C for 1, 3, 5, and 10 min are shown in Figure 1. The optimal condition for the SWE of blueberries was 130 °C for 3 min, which produced an anthocyanin content of 0.47 ± 0.03 mg/g of fresh weight (FW) (mean ± SD) and a malvidin-3-galactoside content of 0.18 ± 0.02 mg/g of FW. Seen at 110 °C and 130 °C, the content of anthocyanin increased and then decreased with an increasing extraction time over 3 min. Conversely, the anthocyanin content decreased exponentially with an increasing extraction time at 150 °C and 170 °C temperatures.

The anthocyanin components of blueberry SWE extracts were identified by high-performance liquid chromatography-mass spectrometry (HPLC-MS) analysis (Table 1). Thirteen anthocyanins were detected: cyanidin-3-arabinoside, cyanidin-3-galactoside, cyanidin-3-glucoside, delphinidin-3-galactoside, delphinidin-3-glucoside, peonidin-3-galactoside, delphindin-3-arabinoside, petunidin-3-arabinoside, malvidin-3-galactoside, petunidin-3-galactoside, petunidin-3-glucoside, malvidin-3-glucoside, and malvidin-3-arabinoside.

Table 2 indicates that the concentration of anthocyanin at the optimal SWE condition was about 4.5 times higher than that of pressed juice (0.04 ± 0.00 mg/g FW) and about 1.5 times higher than that for the extract obtained using hot water (0.12 ± 0.01 mg/g FW). The subcritical water at 100 °C or higher had a lower dielectric constant than that of water at 60 °C, thus affecting the extraction efficiency of anthocyanin.

**Figure 2:**
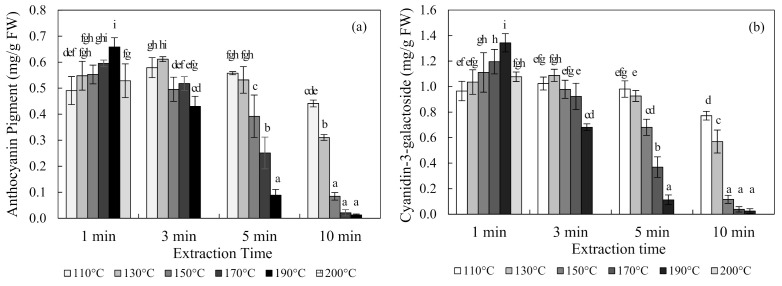
Anthocyanin pigment (**a**) and cyanidin-3-galactoside (**b**) concentrations of chokeberry extracts obtained using subcritical-water extraction (SWE) as analyzed by the pH differential method and high-performance liquid chromatography (HPLC) analysis. Means in bars with same letters are not significantly different according to Duncan’s test at *p* < 0.05. Data are mean and SD values (*n* = 3). FW (fresh weight).

The content of anthocyanin in chokeberries obtained using SWE at 110 °C, 130 °C, 150 °C, 170 °C, 190 °C, and 200 °C for 1, 3, 5, and 10 min are shown in Figure 2. The optimal condition for the SWE of chokeberries was 190 °C for 1 min, which produced an anthocyanin content of 0.66 ± 0.04 mg/g FW (mean ± SD) and a cyanidin-3-galactoside content of 1.34 ± 0.07 mg/g FW. Occurring at 110 °C and 130 °C, the content of anthocyanin increased and then decreased with an increasing extraction time. Conversely, the anthocyanin content decreased exponentially with an increasing extraction time at 150 °C, 170 °C, and 190 °C temperatures.

The anthocyanin components of chokeberry SWE extracts were identified by high-performance liquid chromatography-mass spectrometry (HPLC-MS) analysis. Anthocyanins based on cyanidin and malvidin were the main ones analyzed in the chokeberry extracts (Table 1). Eight anthocyanins were detected: aglycone cyanidin, cyanidin-3-arabinoside, cyanidin-3-galactoside, cyanidin-3-glucoside, delphinidin-3-galactoside, delphinidin-3-glucoside, delphindin-3-arabinoside, and malvidin-3-arabinoside.

Table 2 indicates that the concentration of anthocyanin at the optimal SWE condition was about 9.5 times higher than that of pressed juice (0.14 ± 0.00 mg/g FW) and about 1.7 times higher than that for the extract obtained using hot water (0.77 ± 0.09 mg/g FW). These findings confirm that the SWE method has the advantages of high extraction efficiency and rapid extraction compared to the conventional extraction methods.

### 3.3. Factors Affecting the Optimum Subcritical-Water Extraction (SWE) Condition for Anthocyanins

Anthocyanin yield following extraction is highly affected by various extraction conditions such as temperature and time. The optimal conditions for the extraction of blueberry (malvidin-3-galactoside, 130 °C-3 min) and chokeberry (cyanidin-3-galactoside, 190 °C-1 min) were different. The anthocyanin of the blueberries and chokeberries influenced the optimal condition for subcritical-water extraction (SWE), for which there are several possible reasons. The first is that the solubility of anthocyanins depend on their structure (Table 1). The presence of more methoxy and hydroxyl functional groups in the basic skeleton of anthocyanin will result in a lower solubility. Cyanidin-3-galactoside has four hydroxyl groups in the basic skeleton ring, making it need a higher optimum temperature than malvidin-3-galactoside, which has five methoxy and hydroxyl groups [20,21]. As the temperature increased, the polarity of the water decreased. Occurring at a higher temperature, nonpolar compounds would be dissolved and extracted in a subcritical water state. Regarding the stability of anthocyanins when using the SWE, the results showed that a longer optimum extraction time (malvidin-3-galactoside, 3 min) was more stable than cyanidin-3-galactoside (1 min) at high temperatures.

**Figure 3:**
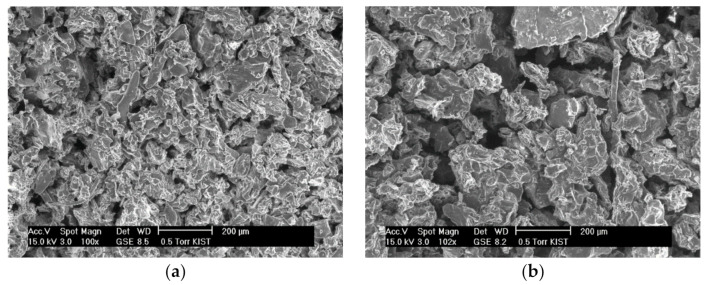
Scanning electron microscopy (SEM) images of blueberry (**a**), and chokeberry (**b**) powder.

The contact area between the solvent and the blueberry and chokeberry samples also could be an influencing factor. The blueberries and chokeberries were dried and pulverized before being used in SWE and scanning electron microscopy (SEM) images of the blueberry and chokeberry powders used for extraction were obtained (Figure 3). The particle size in the extraction of the powders affects extraction efficiency, with the interaction of a chokeberry sample with the solvent being lower when the particles are larger. The optimal extraction temperature of the chokeberries was high to increase the interaction between the solvent and chokeberry powder. Since water at higher temperatures has more energy and a higher diffusion rate, it increases interactions between the solute and solvent. Powders with small particles also have been reported to facilitate diffusion into the plant matrix and provide a greater surface area to provide larger solute and solvent contact [22]. Additionally, the optimal extraction time of chokeberries was relatively short because anthocyanin is sensitive to heat and so degrades when it is exposed to high temperatures for a long time.

### 3.4. Effect of Extraction pH

Anthocyanins are stable in acid, so extraction also was performed using 1% citric acid as the solvent under the optimal condition (130 °C for 3 min for blueberries, 190 °C for 1 min for chokeberries). The anthocyanin content (0.50 ± 0.04 mg/g fresh weight (FW)) and malvidin-3-glactoside content (0.20 ± 0.01 mg/g FW) obtained using 1% citric acid as the solvent were both higher than the content obtained using subcritical-water extraction (SWE) (0.47 ± 0.03 mg/g FW and 0.18 ± 0.02 mg/g FW, respectively) from blueberries. The anthocyanin content (2.52 ± 0.47 mg/g FW) and cyanidin-3-glactoside content (3.77 ± 0.08 mg/g FW) obtained using 1% citric acid as the solvent were both about three times higher than the contents obtained using SWE (0.66 ± 0.04 mg/g FW and 1.34 ± 0.07 mg/g FW, respectively) from chokeberries. This result is consistent with a previous study finding that the anthocyanin extraction efficiency for grape skin increased when using SO_2_ (sulfur dioxide) as the extraction solvent [12]. Another study found that a lower pH resulted in less heat-related damage to anthocyanin [23]. It also has been reported that a lower pH will increase the storage period of anthocyanin [24]. Anthocyanin is a flavylium cation in an acidic condition, which is more stable than in other conditions [6]. Therefore, it was concluded that the extraction efficiency when using 1% citric acid was higher than when using water. The pH decreased by about 1.30 for the extraction performed at 190 °C (the pH was 3.78 with water and 2.48 with 1% citric acid). Water at a higher temperature also exhibits a better dissociation reaction, and a solvent has a lower pH at a higher temperature [25]. During our experiment, we used 1% citric acid (which is an organic acid) to reduce the pH of the solvent, which increased the extraction efficiency of the anthocyanin. Citric acid was used for the extraction of the berries since it is edible, and malic acid, tartaric acid, acetic acid, and ascorbic acid also could be used as extraction solvents. Regarding the pH range, using pH values from 1 to 4 will result in the anthocyanin remaining stable, but the pH should be at least 2 for the extract to be edible [24].

### 3.5. Effect of Flavonoids Content and Antioxidant Activity

**Figure 4:**
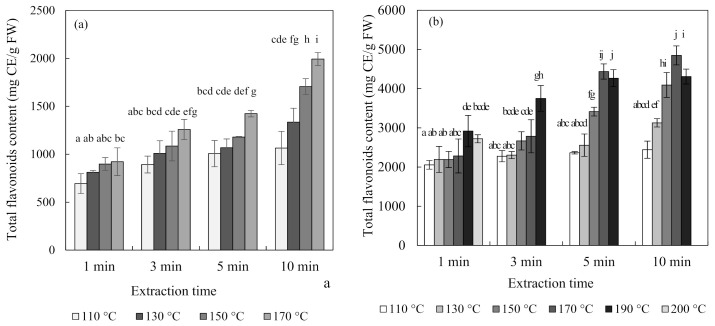

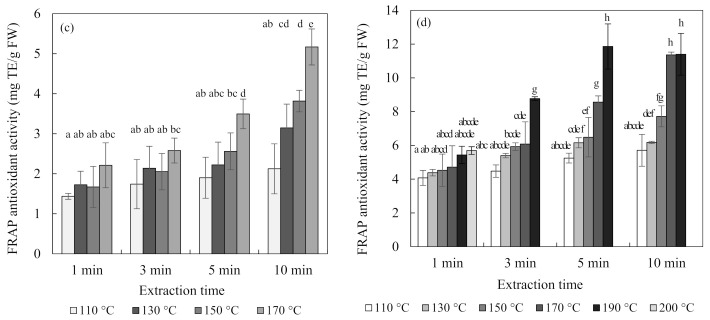
Total flavonoid content and ferric-reducing antioxidant power (FRAP) antioxidant activity of blueberry (**a**,**c**), and chokeberry (**b**,**d**) extracts according to the extraction time and temperature in subcritical-water extraction (SWE) (*n* = 3). TE (trolox equivalent), FW (fresh weight), CE (catechin equivalent). Means in bars with same letters are not significantly different according to Duncan’s test at *p* < 0.05.

Figure 4 shows the total flavonoid contents and antioxidant activity of blueberry and chokeberry extracts and, in the subcritical-water extraction (SWE) condition, both increased continuously with temperature and time. The extraction temperature had a positive effect on the extraction of phenolic compounds such as flavonoids [26,27]. Regarding the case of the total flavonoid content of the chokeberry extract, it decreased at 190 °C for 5 min and 10 min.

Additionally, the changes in the ferric-reducing antioxidant power (FRAP) antioxidant activities of the extracts were similar to those of the total flavonoid contents. While the total flavonoid contents and antioxidant activity showed similar tendencies, the total anthocyanin content did not. The content of anthocyanin tended to decrease with an increasing extraction temperature and time. Ju et al. [12] found similar results for anthocyanin extracted from grape skin using SWE, with the anthocyanin content decreasing but the antioxidant activity increasing as the extraction temperature increased.

There are two possible reasons for these results. The first is the effect of substances other than anthocyanin. Flavonoids include not only anthocyanin but also flavanol species such as catechin and proanthocyanin, as well as quercetin. Blueberries and chokeberries contain many other compounds besides anthocyanin. They have a higher content of polymeric procyanidin, and they contain many antioxidant flavonoids, such as chlorogenic acid, (-)-epicatechin, and quercetin [28]. The second possible reason is the effects of the degradation compounds of anthocyanin [29,30]. Anthocyanin undergoes degradation at high temperatures via weakening of the structure of its skeleton ring. The separated materials mainly include coumaric acid, gallic acid, and benzoic acid. Since all of these substances have antioxidant abilities, the antioxidant ability could be increased even though the content of anthocyanin is decreased at high temperatures. Therefore, the extraction of components other than anthocyanin at high temperatures would affect the tendency for the total flavonoid content and antioxidant activity to increase.

## 4. Conclusions

To conclude, this study determined the optimal extraction conditions for the subcritical-water extraction (SWE) of anthocyanin from blueberries and chokeberries and compared the performance using conventional extraction methods. The anthocyanin yields using SWE were higher than when using hot-water extraction or a pressed-juice extract. SWE is a faster and more-efficient method for extracting anthocyanin from blueberries and chokeberries than conventional extraction methods. This study demonstrates the feasibility of applying SWE to the food processing industry. This method can be easily implemented on an industrial scale.

## Figures and Tables

**Table 1:** Identification of anthocyanins detected by high-performance liquid chromatography-mass spectrometry in blueberry, and chokeberry extracts.

Fruits	Peak	Retention Time (min)	[M]+ (m/z)	Anthocyanin	Chemical Structure	R^1^	R^2^	OR^3^
Blueberry	1	9.931	419.1817	cyanidin-3-arabinoside	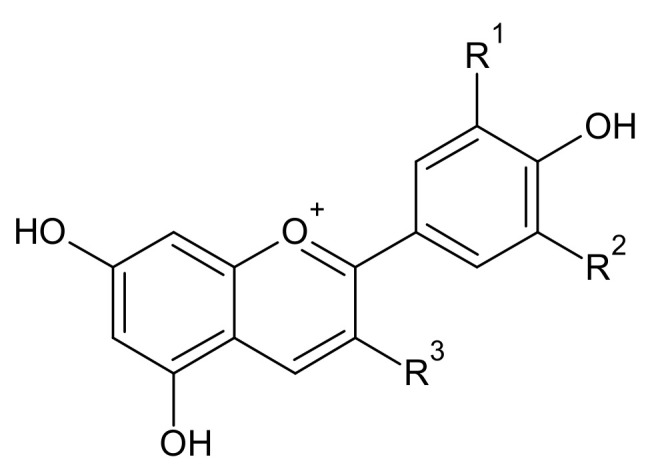	H	OH	arabinose
	2	10.022	449.1723	cyanidin-3-galactoside		H	OH	galactose
	3	10.333	449.177	cyanidin-3-glucoside		H	OH	glucose
	4	10.881	465.1002	delphinidin-3-galactoside		OH	OH	galactose
	5	11.287	465.1007	delphinidin-3-glucoside		OH	OH	glucose
	6	11.903	463.2499	peonidin-3-galactoside		H	OMe	galactose
	7	12.191	435.0865	delphindin-3-arabinoside		OH	OH	arabinose
	8	13.556	449.1049	petunidin-3-arabinoside		OH	OMe	arabinose
	9	15.126	493.0985	malvidin-3-galactoside		OMe	OMe	galactose
	10	16.766	479.1156	petunidin-3-galactoside		OH	OMe	galactose
	11	16.877	479.1251	petunidin-3-glucoside		OH	OMe	glucose
	12	17.101	493.1353	malvidin-3-glucoside		OMe	OMe	glucose
	13	20.697	463.2991	malvidin-3-arabinoside		OMe	OMe	arabinose
Chokeberry	1	0.918	287.110	cyanidin		H	OH	
	2	9.928	419.160	cyanidin-3-arabinoside		H	OH	arabinose
	3	10.016	449.152	cyanidin-3-galactoside		H	OH	galactose
	4	10.338	449.156	cyanidin-3-glucoside		H	OH	glucose
	5	10.874	465.081	delphinidin-3-galactoside		OH	OH	galactose
	6	11.281	465.080	delphinidin-3-glucoside		OH	OH	glucose
	7	12.182	435.079	delphindin-3-arabinoside		OH	OH	arabinose
	8	20.696	463.28	malvidin-3-arabinoside		OMe	OMe	arabinose

**Table 2:** Comparison of the efficiency of extracting anthocyanin from blueberries and chokeberries according to the extraction conditions (*n* = 3). Fresh weight (FW). Means in a row followed by same superscript letters are not significantly different according to Duncan’s test at *p* < 0.05. Data are mean and SD values.

Fruits	Anthocyanin	Extraction Method (mg/g FW)		
		Subcritical Water	Pressed Juice	Hot Water
Blueberry	Anthocyanin pigment	0.47 ± 0.03 ^a^	0.12 ± 0.02 ^c^	0.37 ± 0.05 ^b^
	Malvidin-3-galactoside	0.18 ± 0.02 ^a^	0.04 ± 0.00 ^c^	0.12 ± 0.01 ^b^
Chokeberry	Anthocyanin pigment	0.66 ± 0.04 ^a^	0.08 ± 0.02 ^c^	0.49 ± 0.03 ^b^
	Cyanidin-3-galactoside	1.34 ± 0.07 ^a^	0.14 ± 0.00 ^c^	0.77 ± 0.09 ^b^

## Data Availability

Supplementary data associated with this article can be found.

## Supplementary Materials

The following are available online at https://www.mdpi.com/2304-8158/10/3/527/s1, Figure S1: HPLC chromatograms of the extracts obtained from blueberries; Malvidin-3-galactoside (A), and chokeberries; cyanidin-3-galactoside (B), Figure S2: Spectral characteristics of the extracts in buffers at pH 1 (solid line) and pH 4.5 (dashed line). Figure S3: Relationship between the pH differential method and the HPLC method for the blueberry (A), and chokeberry (B) extracts.
