# Supplementary material for: Anthocyanin Structure and pH Dependent Extraction Characteristics from Blueberries (Vaccinium corymbosum) and Chokeberries (Aronia melanocarpa) in Subcritical Water State
Source: Foods. 2021 Mar 3;10(3):527. doi: 10.3390/foods10030527 (PMC8000843; doi:10.3390/foods10030527)
Supplement: Supplementary file 1 [file foods-10-00527-s001.pdf]

Supplementary Material

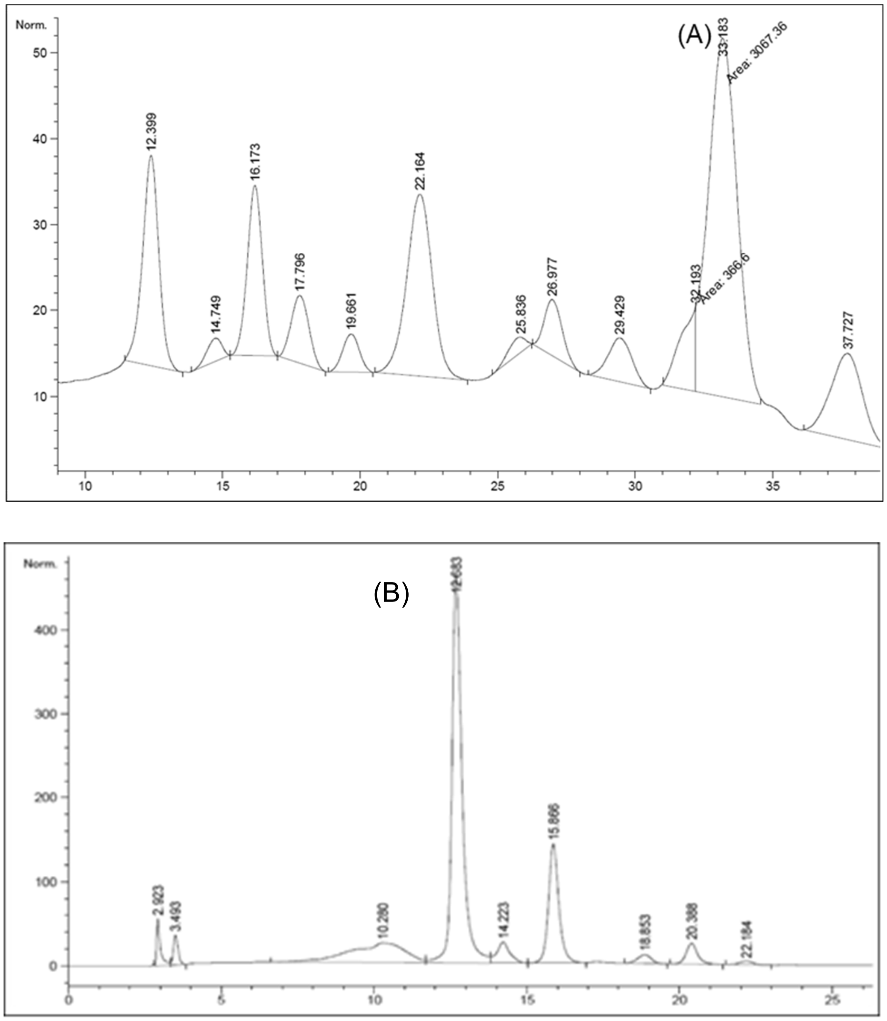

Figure S1. HPLC chromatograms of the extracts obtained from blueberries; Malvidin-3-galactoside (A), and chokeberries; cyanidin-3-galactoside (B).

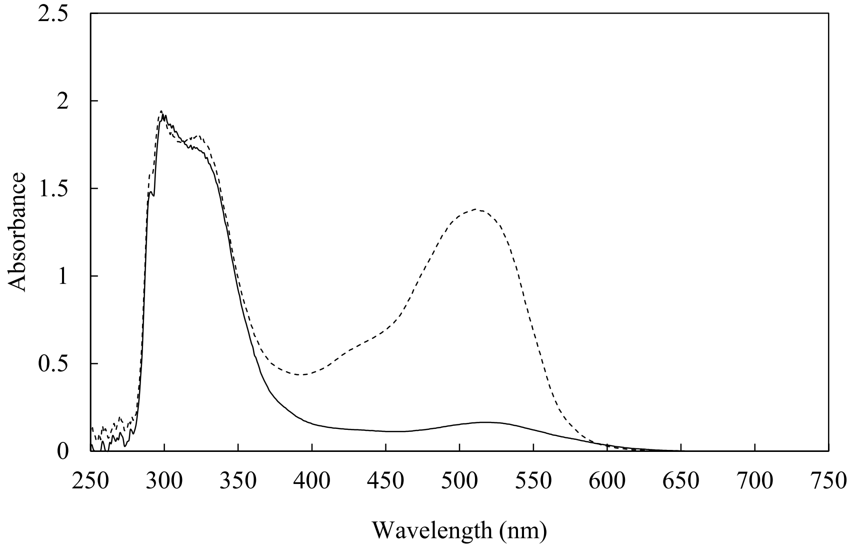

Figure S2. Spectral characteristics of the extracts in buffers at pH 1.0 (solid line) and pH 4.5 (dashed line).

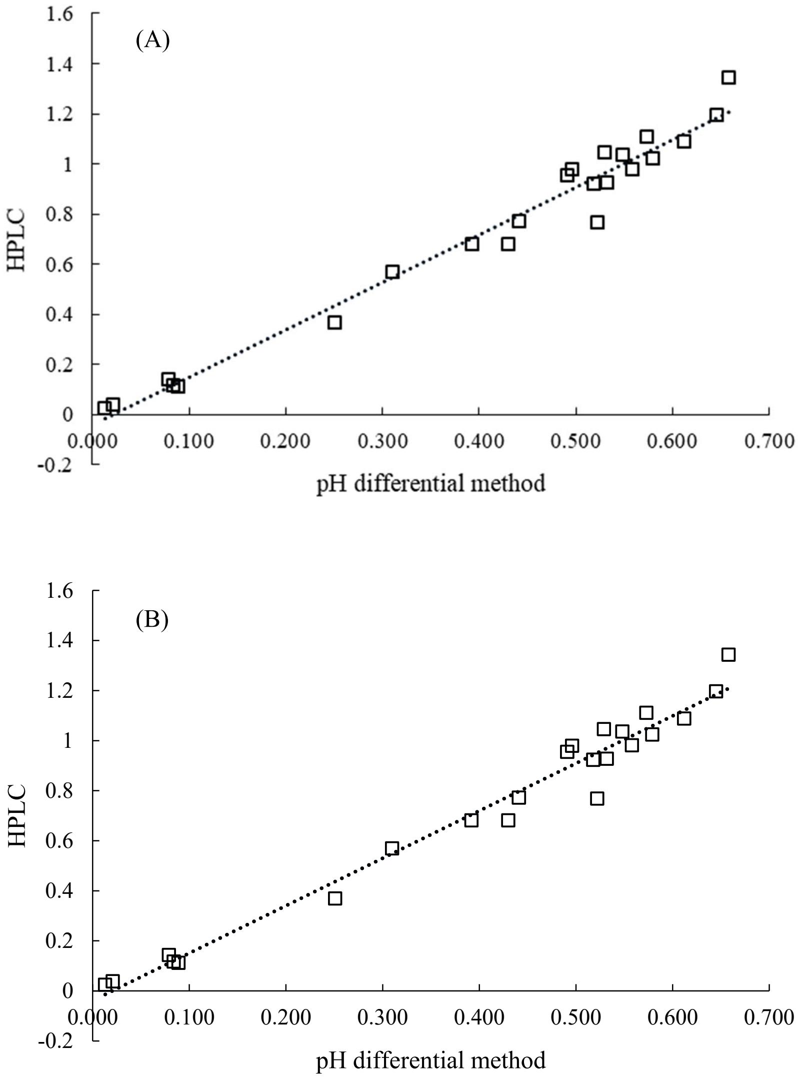

Figure S3. Relationship between the pH differential method and the HPLC method for the blueberry (A), and chokeberry (B) extracts.
